# Supplementary material for: A missense mutation in the barley Xan-h gene encoding the Mg-chelatase subunit I leads to a viable pale green line with reduced daily transpiration rate
Source: Plant Cell Rep. 2024 Sep 29;43(10):246. doi: 10.1007/s00299-024-03328-2 (PMC11439855; doi:10.1007/s00299-024-03328-2)
Supplement: Supplementary file 3 — Supplementary file3 (DOCX 69 KB) [file 299_2024_3328_MOESM3_ESM.docx]

**Table S2**. A subset of nuclear genes that code for proteins located in the chloroplast and are differentially expressed in xan-h.chli-1 and Xan-h leaves under greenhouse conditions.

| **Gene code**  **(**HORVU.MOREX.r3) | **Gene name** | **Biological process/function** | **LogFC** | **Ref** |
| --- | --- | --- | --- | --- |
| Up-regulated genes in *xan-h.chli-1* *vs* *Xan-h* | | | | |
| *4HG0343330* | *DNA glycosylase superfamily protein (ATNTH1)* | Repair of oxidative DNA damages | 7,59 | (Gutman and Niyogi 2009) |
| *6HG0540480* | *Lipoxygenase 2 (LOX2)* | Jasmonic Acid-mediated stress response | 6,94 | (Kaur et al. 2023) |
| *7HG0639230* | *Lipoxygenase 2 (LOX2)* | Jasmonic Acid-mediated stress response | 6,12 | (Kaur et al. 2023) |
| *4HG0392440* | *Chaperon DnaJ-domain superfamily (DJC24)* | Recruit Hsp70 to perform tissue specific function | 2,78 | (Chiu et al. 2013) |
| *5HG0489830* | *Heat Shock Protein 21 (HSP21)* | Required for seedling and chloroplast development under heat stress | 2,05 | (Sedaghatmehr, Mueller-Roeber, and Balazadeh 2016) |
| *5HG0420500* | *Lipoxygenae 2 (LOX2)* | Jasmonic Acid-mediated stress response | 1,58 | (Chiu et al. 2013) |
| *3HG0323270* | *Alpha/beta-hydrolases superfamily (DALL4)* | Jasmonic acid biosynthesis | 1,51 | (Ruduś et al. 2014) |
| *4HG0394970* | *Allene oxide synthase (CYP74A)* | Jasmonic acid biosynthesis | 1,35 | (Oshita et al. 2023) |
| *2HG0097390* | *Cystathionine beta-synthase family protein (CBSX1)* | Stabilize cellular redox homeostasis and modulate plant development via regulation of Thioredoxin systems | 1,15 | (Ok, Yoo, and Shin 2012) |
| *7HG0738610* | *Thylakoid processing peptidase (TTP1)* | Thylakoidal processing peptidase that removes signal sequences from proteins transported into the thylakoid lumen | 1,12 | (Hsu et al. 2011) |
| *7HG0749460* | *Thioredoxin M-type 4 (TRXM4)* | Prokaryotic thioredoxin involved in the response to oxidative stress and control of cyclic electron transport and Calvin cycle. | 0,86 | (Okegawa and Motohashi 2015) |
| *5HG0482040* | *Chlorophyll A-B binding family protein (ELIP1)* | Prevent excess accumulation of free chlorophyll by inhibiting the entire chlorophyll biosynthesis pathway and hence prevent photooxidative stress. | 0,76 | (Rizza et al. 2011) |
| *1HG0053910* | *Plastid Transcriptionally Active Chromosome 18 (PTAC18)* | Associated with the PEP and chloroplast transcription activity and chloroplast biogenesis | 0,75 | (Pfalz et al. 2006) |
| *6HG0595460* | *RmlC-like cupins superfamily protein (TRR14)* | Overexpression leads to trehalose resistance, drought and stress tolerance. *trr14* mutants have reduced seed germination, root length, survival rate and chlorophyll content under stress conditions. | 0,71 | (Aghdasi, Fazli, and Bagherieh 2012) |
| *2HG0165450* | *Arogenate oxigenase 6 (ADT6)* | A plastid-localized arogenate dehydratase involved in phenylalanine biosynthesis. | 0,69 | (Apuya et al. 2002) |
| *6HG0576310* | *Raspberry 3 (RSY3)* | chloroplast biogenesis and seedling development | 0,60 | (Apuya et al. 2002) |
| Down-regulated genes in *xan-h.chli-1* *vs Xan-h* | | | | |
| *7HG0739190* | *Cryptochrome 3 (CRY3)* | Bind flavin adenine dinucleotide and DNA. It is likely to act as photoreceptor | -0,60 | (Göbel et al. 2017) |
| *7HG0738860* | *Peptidyl-prolyl cis-trans isomerase (FKBP13)* | FKBP13 is located in chloroplast thylakoid lumen and possess peptidyl–prolyl cis/trans isomerase (PPIase) activity. | -0,68 | (Ingelsson et al., 2009) |
| *7HG0689310* | *Late embryogenesis abundant hydroxyproline-rich glycoprotein family (LEA)* | Function in normal plant growth and development, and in protecting cells from abiotic stress | -0,81 | (Hong-Bo, Zong-Suo and Ming-An, 2005) |
| *5HG0533290* | *ACYL ACTIVATING ENZYME 16 (AAE16)* | Fatty acid biosynthetic process | -1.06 | (Tjellström et al. 2013) |
| *4HG0418700* | *RUBISCO ASSEMBLY FACTOR 2 (RAF2)* | Protein involved in Rubisco assembly that also mediates Abscisic acid-dependent stress response. | -1.28 | (Fristedt et al. 2018) |
| *7HG0712530* | *Alpha amylase family protein (BE1)* | Putative glycoside hydrolase localized in plastids, plays crucial roles during embryogenesis and carbohydrate metabolism | -4,01 | (Wang et al. 2010) |
| *3HG0228020* | *Sulfoquinovosyldiacylglycerol 2 (SQD2)* | Involved in sulfolipid biosynthesis | -4.07 | (Okazaki et al. 2013) |
| *7HG0689300* | *Thioredoxin family protein* | Change in response to H2O2. | -10.06 | (Peltier et al. 2004) |

**References**

Aghdasi, Mahnaz, Fariba Fazli, and Mohammad Bagher Bagherieh. 2012. ‘Cloning and Expression Analysis of Arabidopsis TRR14 Gene under Salt and Drought Stress’. *Journal of Cell and Molecular Research* 4 (1): 1–10. <https://doi.org/10.22067/JCMR.V4I1.12269>.

Apuya, Nestor R., Ramin Yadegari, Robert L. Fischer, John H. Harada, and Robert B. Goldberg. 2002. ‘RASPBERRY3 Gene Encodes a Novel Protein Important for Embryo Development’. *Plant Physiology* 129 (2): 691–705. https://doi.org/10.1104/PP.004010.

Chiu, Chi Chou, Lih Jen Chen, Pai Hsiang Su, and Hsou min Li. 2013. ‘Evolution of Chloroplast J Proteins’. *PLOS ONE* 8 (7): e70384. <https://doi.org/10.1371/JOURNAL.PONE.0070384>.

Fristedt, Rikard, Chen Hu, Nicole Wheatley, Laura M. Roy, Rebekka M. Wachter, Linda Savage, Jeremy Harbinson, et al. 2018. ‘RAF2 Is a RuBisCO Assembly Factor in Arabidopsis Thaliana’. *The Plant Journal* 94 (1): 146–56. https://doi.org/10.1111/TPJ.13849.

Gutman, Benjamin L., and Krishna K. Niyogi. 2009. ‘Evidence for Base Excision Repair of Oxidative DNA Damage in Chloroplasts of Arabidopsis Thaliana’. *Journal of Biological Chemistry* 284 (25): 17006–12. <https://doi.org/10.1074/JBC.M109.008342>.

Göbel, Tanja, Stefan Reisbacher, Alfred Batschauer, and Richard Pokorny. 2017. ‘Flavin Adenine Dinucleotide and N5,N10-Methenyltetrahydrofolate Are the in Planta Cofactors of Arabidopsis Thaliana Cryptochrome 3’. *Photochemistry and Photobiology* 93 (1): 355–62. <https://doi.org/10.1111/PHP.12622>.

Hong-Bo, Shao, Liang Zong-Suo, and Shao Ming-An. 2005. ‘LEA Proteins in Higher Plants: Structure, Function, Gene Expression and Regulation’. *Colloids and Surfaces B: Biointerfaces* 45 (3–4): 131–35. https://doi.org/10.1016/J.COLSURFB.2005.07.017.

Hsu, Shih Chi, Joshua K. Endow, Nicholas J. Ruppel, Rebecca L. Roston, Amy J. Baldwin, and Kentaro Inoue. 2011. ‘Functional Diversification of Thylakoidal Processing Peptidases in Arabidopsis Thaliana’. *PLOS ONE* 6 (11): e27258. <https://doi.org/10.1371/JOURNAL.PONE.0027258>.

Ingelsson, Bjorn, Alexey, Shapiguzov, Thomas, Kieselbach, Alexander V, Vener. 2009. Peptidyl-prolyl isomerase activity in chloroplast thylakoid lumen is a dispensable function of immunophilins in Arabidopsis thaliana. *Plant Cell Physiol.* 50(10): 1801-14. doi: 10.1093/pcp/pcp122.

Kaur, Diljot, Sonia Dorion, Souleimen Jmii, Laurent Cappadocia, Jacqueline C. Bede, and Jean Rivoal. 2023. ‘Pseudophosphorylation of Arabidopsis Jasmonate Biosynthesis Enzyme Lipoxygenase 2 via Mutation of Ser600 Inhibits Enzyme Activity’. *Journal of Biological Chemistry* 299 (3): 102898. <https://doi.org/10.1016/J.JBC.2023.102898>.

Ok, Sung Han, Kyoung Shin Yoo, and Jeong Sheop Shin. 2012. ‘CBSXs Are Sensor Relay Proteins Sensing Adenosine-Containing Ligands in Arabidopsis’. *Plant Signaling & Behavior* 7 (6): 664–67. <https://doi.org/10.4161/PSB.19945>.

Okazaki, Yozo, Hitomi Otsuki, Tomoko Narisawa, Makoto Kobayashi, Satoru Sawai, Yukiko Kamide, Miyako Kusano, Toshio Aoki, Masami Yokota Hirai, and Kazuki Saito. 2013. ‘A New Class of Plant Lipid Is Essential for Protection against Phosphorus Depletion’. *Nature Communications 2013 4:1* 4 (1): 1–10. https://doi.org/10.1038/ncomms2512.

Okegawa, Yuki, and Ken Motohashi. 2015. ‘Chloroplastic Thioredoxin m Functions as a Major Regulator of Calvin Cycle Enzymes during Photosynthesis in Vivo’. *The Plant Journal* 84 (5): 900–913. https://doi.org/10.1111/TPJ.13049.

Oshita, Tomoki, Joongeun Sim, Taufika Islam Anee, Hanako Kiyono, Chihiro Nozu, and Nobuhiro Suzuki. 2023. ‘Attenuation of Negative Effects Caused by a Combination of Heat and Cadmium Stress in Arabidopsis Thaliana Deficient in Jasmonic Acid Synthesis’. *Journal of Plant Physiology* 281 (February): 153915. <https://doi.org/10.1016/J.JPLPH.2023.153915>.

Peltier, Jean Benoit, A. Jimmy Ytterberg, Qi Sun, and Klaas J. Van Wijk. 2004. ‘New Functions of the Thylakoid Membrane Proteome of Arabidopsis Thaliana Revealed by a Simple, Fast, and Versatile Fractionation Strategy’. *Journal of Biological Chemistry* 279 (47): 49367–83. https://doi.org/10.1074/JBC.M406763200.

Pfalz, Jeannette, Karsten Liere, Andrea Kandlbinder, Karl Josef Dietz, and Ralf Oelmüller. 2006. ‘PTAC2, -6, and -12 Are Components of the Transcriptionally Active Plastid Chromosome That Are Required for Plastid Gene Expression’. *The Plant Cell* 18 (1): 176–97. https://doi.org/10.1105/TPC.105.036392.

Rizza, Annalisa, Alessandra Boccaccini, Irene Lopez-Vidriero, Paolo Costantino, and Paola Vittorioso. 2011. ‘Inactivation of the ELIP1 and ELIP2 Genes Affects Arabidopsis Seed Germination’. *New Phytologist* 190 (4): 896–905. https://doi.org/10.1111/J.1469-8137.2010.03637.X.

Ruduś, Izabela, Haruka Terai, Takafumi Shimizu, Hisae Kojima, Kazuki Hattori, Yuka Nishimori, Hironaka Tsukagoshi, et al. 2014. ‘Wound-Induced Expression of DEFECTIVE IN ANTHER DEHISCENCE1 and DAD1-like Lipase Genes Is Mediated by Both CORONATINE INSENSITIVE1-Dependent and Independent Pathways in Arabidopsis Thaliana’. *Plant Cell Reports* 33 (6): 849–60. https://doi.org/10.1007/S00299-013-1561-8/FIGURES/6.

Sedaghatmehr, Mastoureh, Bernd Mueller-Roeber, and Salma Balazadeh. 2016. ‘The Plastid Metalloprotease FtsH6 and Small Heat Shock Protein HSP21 Jointly Regulate Thermomemory in Arabidopsis’. *Nature Communications 2016 7:1* 7 (1): 1–14. https://doi.org/10.1038/ncomms12439.

Tjellström, Henrik, Merissa Strawsine, Jillian Silva, Edgar B. Cahoon, and John B. Ohlrogge. 2013. ‘Disruption of Plastid Acyl:Acyl Carrier Protein Synthetases Increases Medium Chain Fatty Acid Accumulation in Seeds of Transgenic Arabidopsis’. *FEBS Letters* 587 (7): 936–42. https://doi.org/10.1016/J.FEBSLET.2013.02.021.

Wang, Xingchun, Li Xue, Jiaqiang Sun, and Jianru Zuo. 2010. ‘The Arabidopsis BE1 Gene, Encoding a Putative Glycoside Hydrolase Localized in Plastids, Plays Crucial Roles during Embryogenesis and Carbohydrate Metabolism’. *Journal of Integrative Plant Biology* 52 (3): 273–88. https://doi.org/10.1111/J.1744-7909.2010.00930.X.
